# Insights into the Reactivity of Brookite TiO_2_ Nanorods in Liquid Water from Ab Initio Molecular Dynamics Simulations

**DOI:** 10.1021/acscatal.5c09228

**Published:** 2026-02-09

**Authors:** Lorenzo Agosta, Giuseppe Zollo, Annabella Selloni

**Affiliations:** † Department of Chemistry, Ångström Laboratory, 8097 Uppsala University, 751 21 Uppsala, Sweden; ‡ Department of Chemistry, 6740 Princeton University, Princeton, New Jersey 08542, United States; ¶ Dipartimento di Scienze di Base e Applicate per l’Ingegneria, University of Rome “La Sapienza”, Via A. Scarpa 14−16, 00161 Rome, Italy

**Keywords:** Water reactivity, nanorod, TiO_2_, exciton, facet, ab initio molecular dynamics

## Abstract

Brookite TiO_2_, a rare natural polymorph of TiO_2_, has been reported to be an excellent photocatalyst for the production of hydrogen from water and aqueous alcohol solutions, especially when it is reduced and synthesized in the form of nanorods. Here, we investigate the reactivity of stoichiometric and reduced brookite nanorods in liquid water using ab initio molecular dynamics and hybrid density functional theory calculations. Our simulations show a much higher water dissociation fraction on reduced nanorods than on stoichiometric ones, with an accumulation of the resulting bridging hydroxyls (O_br_H) and terminal hydroxyls (Ti–OH) on different facets of the nanorod. O_br_H groups accumulate preferentially on low-energy (210) facets, where they are stabilized by adjacent reduced Ti (Ti^3+^) sites, while Ti–OH groups prefer to form at the 4-fold coordinated Ti atoms on high-energy (010) facets. This hydroxylation pattern also favors the spatial localization of excited holes on the (010) facets. This coupling between water-induced surface chemistry and charge separation underpins the enhanced photocatalytic activity of brookite nanorods, providing useful information for the design of more efficient TiO_2_-based nanostructures for solar-driven hydrogen evolution.

## Graphical abstract

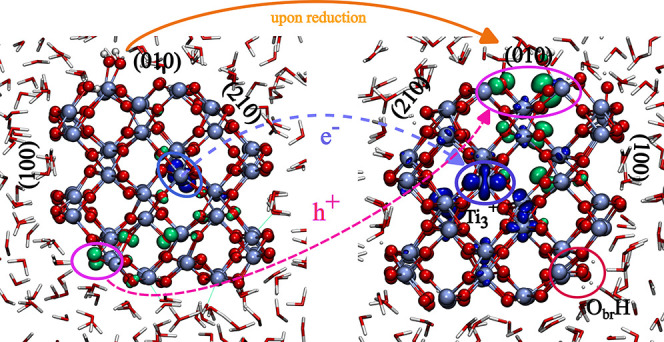

## Introduction

The potential of titanium dioxide (TiO_2_) as a photocatalyst for the production of hydrogen from water and aqueous mixtures has generated broad interest and intensive studies of the structure and reactivity of aqueous TiO_2_ surfaces and nanoparticles over the last decades.^1–5^ These studies have provided important insights into the photocatalytic properties of TiO_2_, including the mechanism of the oxygen evolution reaction, the kinetics of charge carriers, and the roles of defects and polarons.^6–10^ However, there are still significant gaps in the understanding of these systems, such as the behavior of water on less common TiO_2_ surfaces, the interplay between localized and extended surface defects, charge carriers and adsorbates,^5,11,12^ as well as possible synergistic effects arising from different facets in TiO_2_ nanostructures.^13–15^ Indeed, the photocatalytic activity of TiO_2_ depends on the crystalline phase (anatase, rutile, or brookite), as well as on the morphology,^16^ the exposed facets,^17^ and the level of reduction.^18^ While most investigations of TiO_2_ photocatalysis have examined anatase and rutile surfaces and nanoparticles,^6,17,19–25^ recent studies have reported the less common brookite phase as a particularly promising material,^26,27^ often exhibiting higher photocatalytic activity compared to anatase and rutile. In particular, when brookite is synthesized in the form of nanorods, it promotes enhanced hydrogen production from the reforming process of alcohols.^28,29^ This efficiency has been mainly attributed to the character of excess electron states^30^ and the anisotropic geometry of nanorods, which promotes the spatial and temporal separation of photogenerated charge carriers,^29^ but oxygen vacancies and other reducing defects have also been identified as important contributors to the activity of these nanostructures.^31,32^ However, the complex synergy that arises from oxygen vacancies, reduced electronic states, and nanostructured morphology, which is at the origin of the observed enhancement of the photocatalytic activity, remains unclear.

To shed light on this mechanism, here we investigate the structure and spontaneous reactivity of brookite TiO_2_ nanorods in liquid water using ab initio molecular dynamics (AIMD) simulations^33^ complemented by hybrid density functional calculations^34^ of their electronic properties, an approach that has been proven capable of providing reliable mechanistic insight into reactive processes at oxide surfaces and interfaces in previous studies.^5^ Similarly to the rutile and anatase phases, brookite is also composed of distorted TiO_6_ octahedra and includes bulk and surface characteristics of both other phases. Its most stable and abundant (210) surface^35^ is structurally similar to the majority (101) surface of anatase and has been frequently observed in brookite nanorods.^29^ Based on the calculated equilibrium shape of brookite,^35^ for this study we considered stoichiometric and reduced nanorods with the main axis along the [001] direction, exposing low energy (210) and high energy (001) and (100) facets. Our results show that oxygen vacancies dramatically enhance the dissociation fraction of adsorbed water, leading to the formation of facet-selective hydroxyl groups. Bridging hydroxyls accumulate preferentially on (210) facets, where they are stabilized by nearby reduced titanium (Ti^3+^) sites, while terminal Ti–OH^–^ groups form on the more reactive (010) facets. Remarkably, this hydroxylation pattern induces a corresponding spatial segregation of excited holes, which tend to localize on the (010) facets. These findings reveal a previously unrecognized connection between surface hydroxylation and charge separation in TiO_2_ nanostructures, offering new insights into their photocatalytic behavior and possibly leading to new strategies for facet engineering in hydrogen evolution systems based on TiO_2_.

## Results and Discussion

The model of brookite nanorod used in our study is periodic along its main axis (*z*) and has a diameter of ∼1.2 nm (see Figure 1). Although a few times smaller than the typical experimental diameters of 4–6 nm,^28^ this size is sufficient to clearly distinguish between the surface and bulk-like regions of the nanorod. The nanorod exposes (210), (100), and (010) facets, with relative areas proportional to the corresponding surface energies^35,36^ according to the Wulff construction. All facets expose 2-coordinated bridging oxygen atoms, O_*br*_, as well as undercoordinated Ti atoms, notably 5-fold coordinated Ti_5*c*_ atoms on the (100) and (210) facets and 4-fold coordinated Ti_4*c*_ atoms on the (010) facet, which are the most reactive metal sites on the nanorod’s surface.

**Figure 1:**
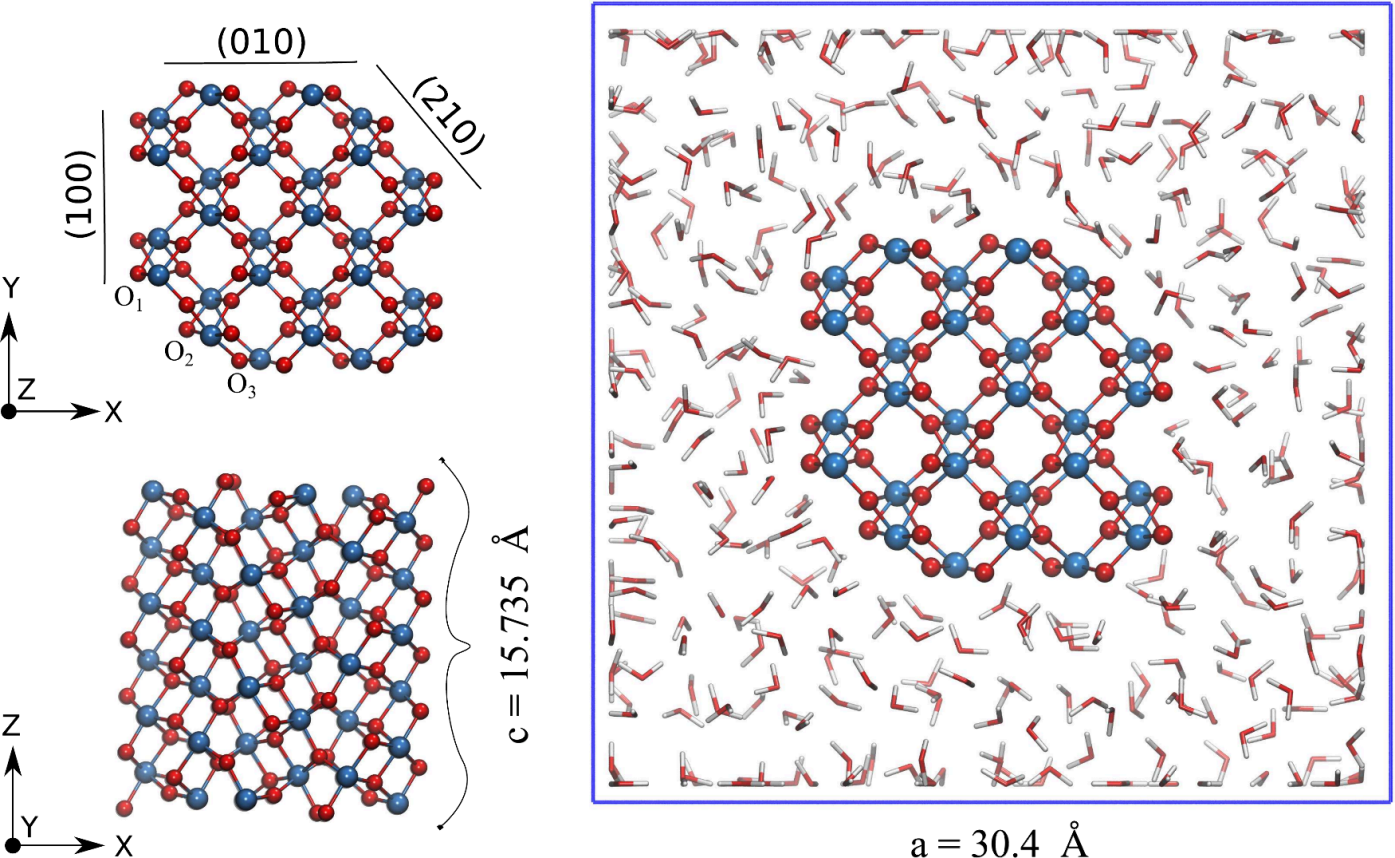
Brookite nanowire model exposing (210), (100), and (010) facets immersed in liquid water. The system is periodic along the [001] (*z*) axis and includes 84 TiO_2_ units per unit cell. The hydrated system contains 360 water molecules at the experimental room temperature density of ∼1 g cm^–3^.

### Water Dissociation at the Nanorod–Water Interface

To shed light on the hydrogen evolution activity, we performed AIMD simulations of stoichiometric and reduced nanorods immersed in liquid water, focusing on their reactivity for water dissociation, which is the first step of water splitting; computational details are given in the “Methods and Models” section of the Supporting Information (SI). In Figure 2, we show the time evolution of the number of hydroxyl groups Ti–OH (terminal) and O_br_H (bridging) on the surface of the stochiometric nanorod during an AIMD trajectory of ∼60 ps, where all interfacial water was initially in molecular form. In the first 30 ps of the simulation, Ti–OH and O_br_H groups are only observed on the (010) facet, where the highly reactive undercoordinated Ti_4*c*_ atoms are present. The numbers of Ti–OH and O_br_H are the same, which confirms that they originate from the dissociation of water. After 30 ps, some hydroxyl groups are also observed on the (100) facet, while only one dissociation event is observed, after about 50 ps, on the (210) facet, which was reported to be reactive in a previous study.^37^ Test calculations on the adsorption of a single water molecule suggest that this difference may originate from the different morphologies and DFT functionals used in our work and in those studies.

**Figure 2:**
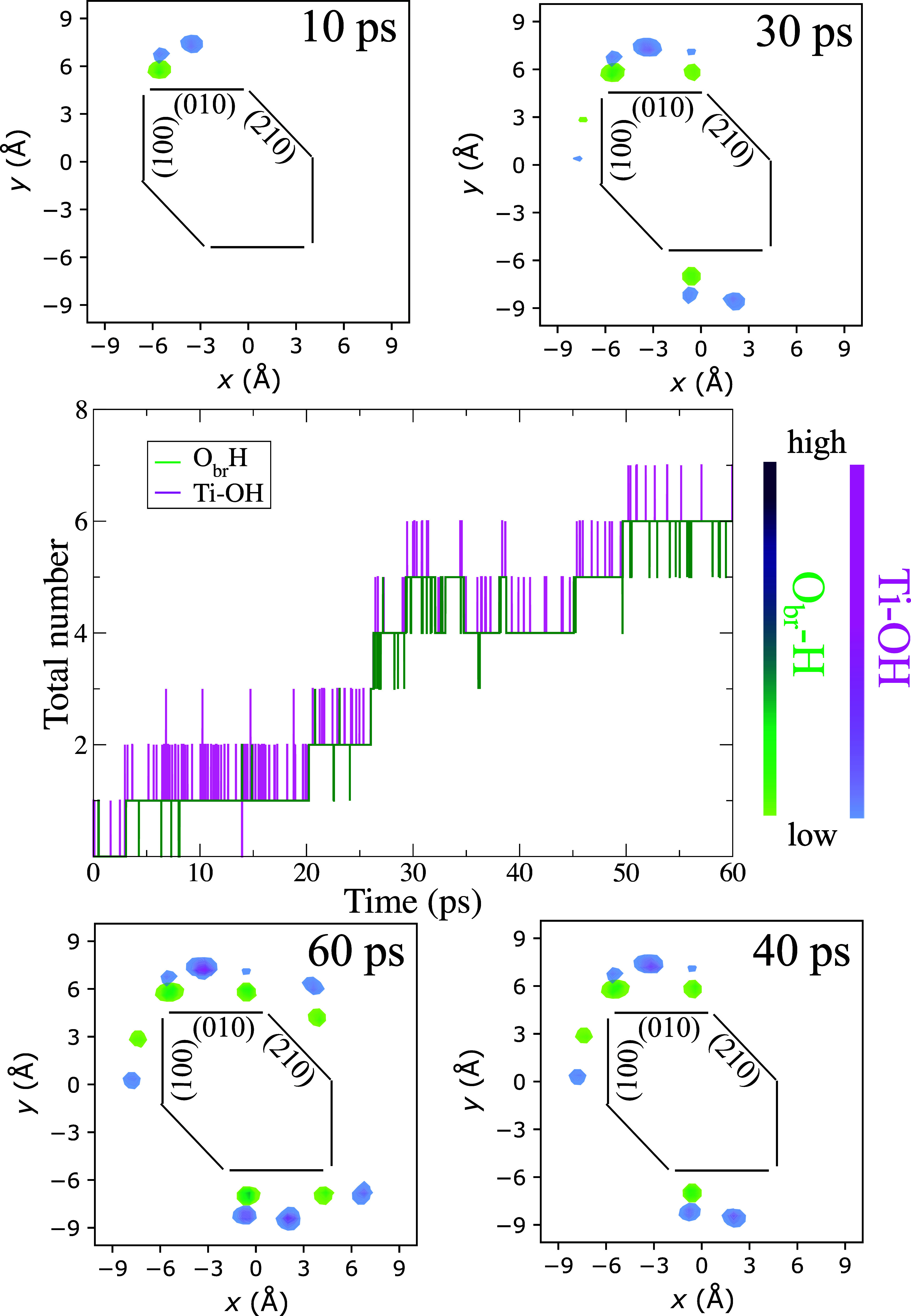
Time evolution of the amount of water dissociation on the different facets of the stoichiometric brookite nanorod in liquid water along a ∼60 ps AIMD simulation. The central panel shows the time evolution of the total number of Ti–OH (terminal) and O_br_H (bridging) hydroxyl groups on the surface of the nanorod. The top and bottom panels show the distribution of Ti–OH and O_br_H groups on the various facets at selected times along the AIMD trajectory. The black lines indicate the positions of the facets.

The small fraction of water dissociation that we find for the stoichiometric nanorod (Figure 2) appears to be incompatible with the high rate of H_2_ production observed experimentally on brookite nanorods. In fact, experiments have found that reduced Ti^3+^ sites associated with oxygen vacancies are essential to promote the catalytic properties of brookite nanorods.^29^ Based on this evidence, we created some surface oxygen vacancies at favorable O_1_ sites on the edges of the nanorod (details of the model are given in the SI) and investigated the reactivity of the reduced structure in water by AIMD.

The results of our simulations are summarized in Figure 3, which illustrates the complex reactivity pattern resulting from the presence of oxygen vacancies (see also Figure S1 in the Supporting Information). We note fast water dissociation in the first 8 ps, which begins at Ti_4*c*_ sites on (010) facets and O_1_ vacancies at the edge between (100) and (210) facets and propagates toward (210) facets. The dissociation of a water molecule at a Ti_4*c*_ surface site on the (010) facet gives rise to a terminal Ti–OH and a bridge O_br_ H hydroxyl. In contrast, the dissociation of a water molecule at an O_1_ vacancy site results in the formation of two bridging O_br_H hydroxyls,^38,39^ which rapidly migrate to neighboring (210) facets by the Grotthuss mechanism for proton hopping.^40^ The amount of water dissociation increases with time, enhancing the density of hydroxyl groups on the (010) facets and triggering the formation of some Ti–OH groups also on the (210) surface. After ∼32 ps, we start noticing some water dissociation at the edge between the (010) and (100) facets (indicated by the appearance of OH densities on the (100) facet), where protons of adsorbed water at Ti sites jump back and forth to neighboring oxygen sites to form O_br_H and Ti–OH groups. The total amount of water dissociation reaches a plateau of ∼28% of the Ti and O_br_ available sites after ∼40 ps, with terminal and bridging hydroxyl groups most abundant on (010) and (210) facets, respectively. Interestingly, the increase in the amount of adsorbed water dissociation in the reduced nanorod compared to the stoichiometric case also induces a slight increase in the density of interfacial water, suggesting a higher hydrophilicity of the reduced surface (see Figure S2 in the Supporting Information).

**Figure 3:**
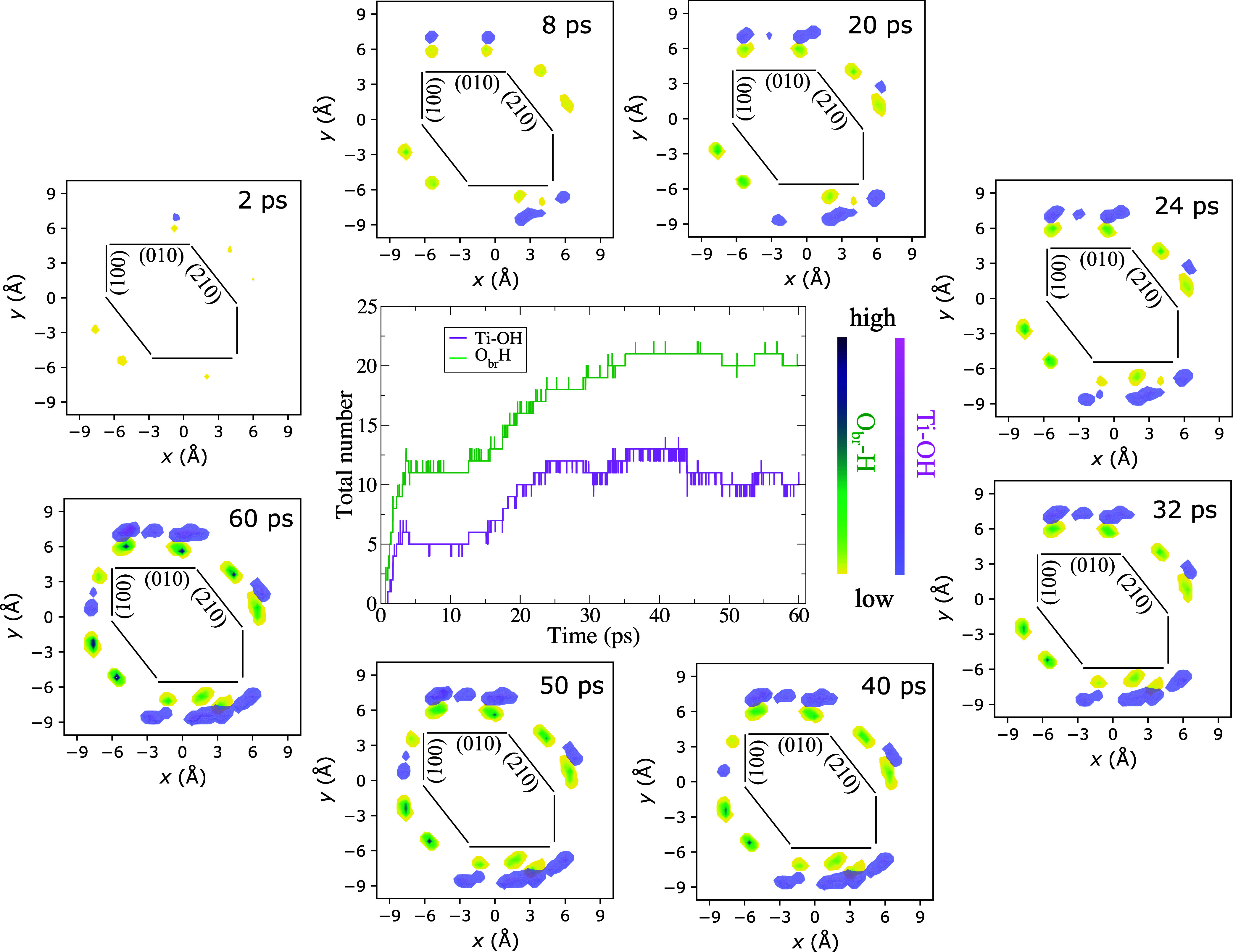
Time evolution of the amount of water dissociation on the different facets of the reduced brookite nanorod in liquid water along a ∼ 60 ps AIMD simulations. The central panel shows the time evolution of the total number of Ti–OH and O_br_H groups on the surface of the nanorod. The surrounding panels show the distribution of Ti–OH and O_br_H groups on the various facets at selected times along the AIMD trajectory. The black lines indicate the positions of the facets.

The enhanced reactivity of the reduced nanorod in comparison to the stoichiometric case provides clear evidence of the crucial role of surface oxygen vacancies in the generation of hydroxyl groups. However, the morphology of the nanorod and the presence of edges are also critical in this process because oxygen vacancies preferentially form at the edge sites, and surface hydroxyls resulting from the dissociation of water at these sites can easily spread to adjacent facets. It is also worth noting that the energy gain obtained by immersing the reduced nanorod in bulk water is much larger than that for its stoichiometric counterpart, as seen by comparing the relative total potential energies (see Figure S3 in the Supporting Information). The adsorption energy of water on the reduced nanorod is about 9 eV lower than on the stoichiometric rod, evidencing a marked stabilization effect driven by hydration.

### Electronic Properties

To obtain information on the water dissociation patterns shown in Figures 2 and 3, we calculated the density of state (DOS) of the stoichiometric and reduced nanorods in water, along with the projected DOS on the undercoordinated atoms of the (010) and (210) facets (Figure 4). The DOS of the reduced structure (red curve) shows the presence of occupied gap states below the conduction band that are associated with the reduced Ti^3+^ ions induced by oxygen vacancies.^41^ In the optimized structure (*T* = 0 K) of the dry nanorod, the gap states are well-localized in the d orbitals of Ti atoms close to the oxygen vacancies (see Figure S4a in the Supporting Information). However, for the reduced nanorod in water, Ti^3+^ ions are present in both the bulk of the nanostructure and below the O_br_ H hydroxyls on the (210) facets (see Figure S4b in the Supporting Information). Some Ti^3+^ ions are also found at the undercoordinated surface Ti sites of (210) facets, and their states have energies lower than those of the bulk states (green curve in Figure 4).

**Figure 4:**
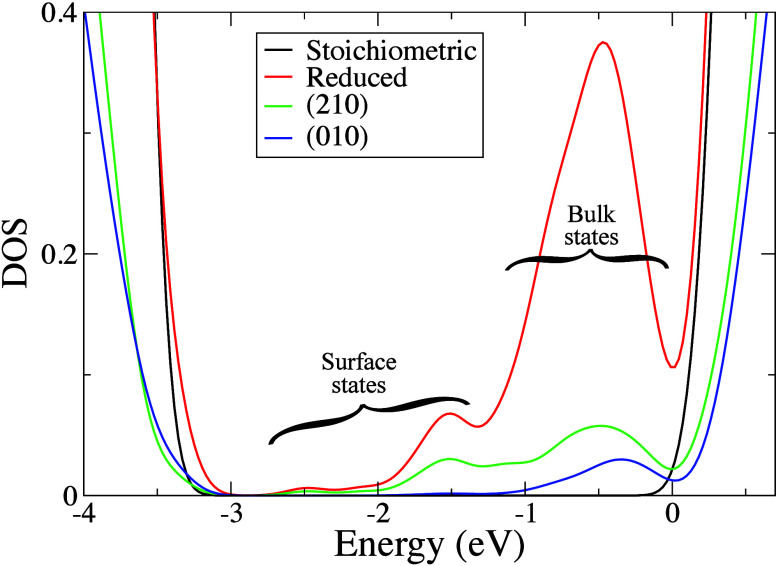
Electronic density of states (DOS) of the hydrated stoichiometric (black) and reduced (red) nanorods, from hybrid B3LYP functional^34^ calculations. Also shown are the projected DOS of undercoordinated O and Ti atoms belonging to the (210) and (010) facets (green and blue lines). The zero energy is set at the Fermi level of the reduced nanorod. A large part of the reduced Ti^3+^ states, in the range 0 to −1 eV, are localized in the inner part of the nanorod (labeled as “bulk states” in the figure), while the “surface states” (i.e., states localized on the surface undercoordinated Ti atoms of the nanorod) are mostly contributed by the (210) facet.

From these results, it appears that the (210) facets are characterized by an accumulation of both O_br_ H hydroxyls and Ti^3+^ ions, as found when the surface of TiO_2_ is hydrogenated.^11,31^ Such abundance of Ti^3+^ sites is consistent with the structural similarity of the brookite (210) surface to anatase (101),^35^ which is known to be the preferred surface for reduction reactions in anatase.^11,42^

### Excited Electrons and Holes

While all the results presented so far refer to the TiO_2_–water system in the electronic ground state, photocatalysis is based on the generation of charge carriers (electron–hole pairs) in the semiconductor by optical absorption, followed by their transfer to adsorbed species on the TiO_2_ surface.^3^ However, accurately describing photoexcited electrons and holes is computationally difficult for a large system like the hydrated nanorod we are considering. In this work, we thus used a simplified approach in which electron–hole pairs are created by promoting an electron from the valence to the conduction band using spin-constrained calculations (see the “Methods and Models” section in the SI). Specifically, for the stoichiometric nanorod the excess electron and hole are introduced by considering the lowest energy triplet spin configuration above the singlet ground state, an approach frequently used to approximate a photoexcited state in previous studies.^41,43–45^ An analogous spin-constrained approach is used to model excited electrons and holes in the reduced nanorod. In all cases, the geometry of the corresponding ground state is used.

Our calculations for the stoichiometric and reduced brookite nanorods under vacuum (at *T* = 0 K) predict that the excited electron and hole are located in the Ti 3d and O 2p states of a few atoms in the core of the nanorod (see Figure S5 in Supporting Information) and form energy levels below the minimum of the conduction band and above the maximum of the valence band, respectively (Figure S6 in the Supporting Information). However, the picture changes when the nanorods are immersed in liquid water (Figure S7 of Supporting Information). In the hydrated stoichiometric structure, the hole is in part attracted to the bridging oxygens of (210) facets, while the excited electron is mainly in the bulk (Figures 5a). In the hydrated reduced nanorod, instead, the hole is located mainly in proximity of the Ti_4*c*_–OH^–^ groups on the (010) facets, while the location of the excited electron varies and is either close to the hole or at distant sites (Figures 5b).

**Figure 5:**
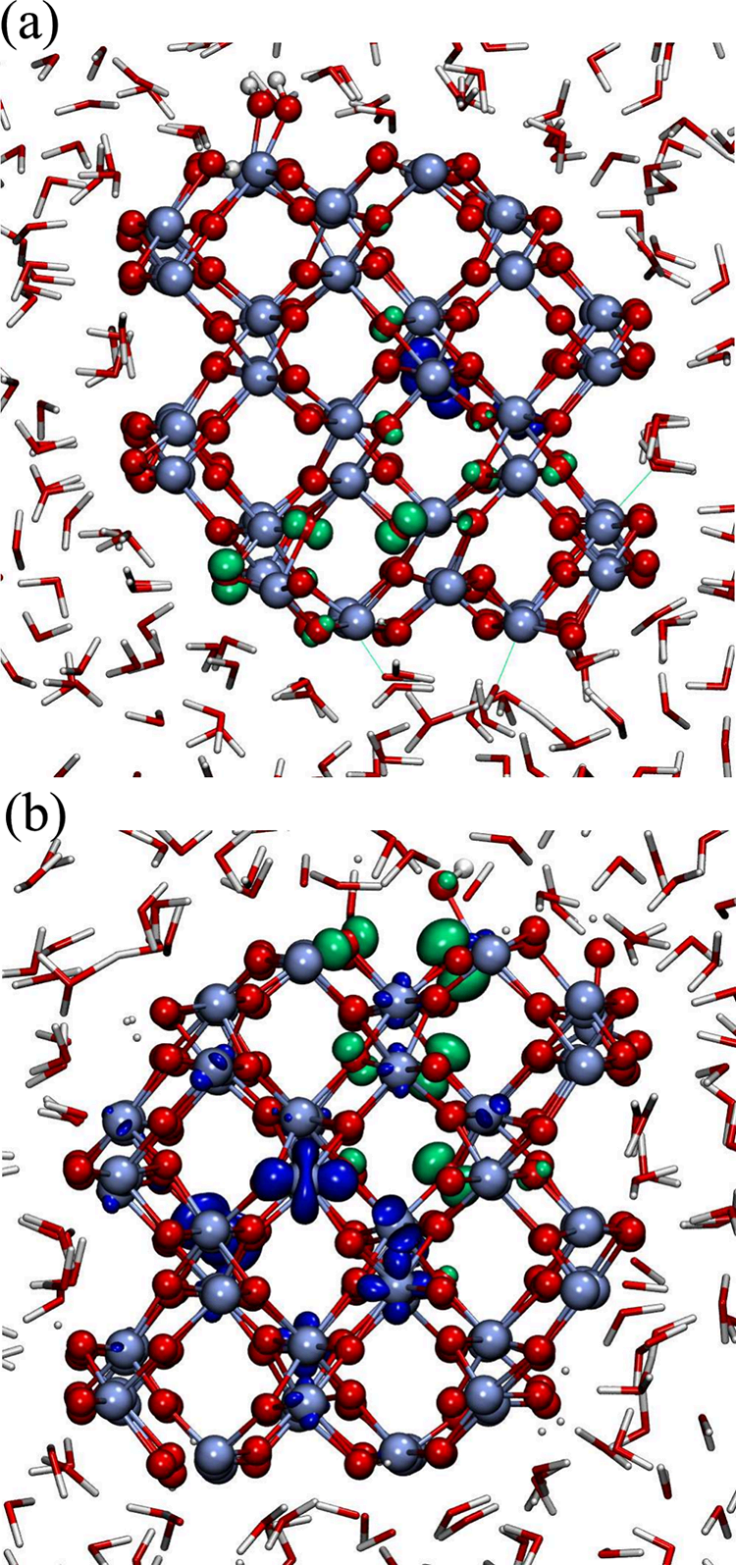
Densities of the excited electron and hole (blue and green isosurface, respectively) in the hydrated (a) stoichiometric and (b) reduced nanorod. The snapshots are representative configurations of the equilibrated systems at room temperature. For the stoichiometric nanorod the hole remains in the bulk or on the (210) facet. When the nanorod is reduced the hole is mainly localized on the bridging oxygens in proximity of the Ti_4*c*_–OH groups of the (010) facet.

Overall, the results of our simulations suggest that the accumulation of the O_br_H and Ti–OH groups in different facets, (210) and (010) respectively, of the nanorod (Figure 3) promotes the localization and stabilization of the reduced Ti^3+^ states close to the (210) facets, while excited holes are preferentially formed on the (010) facet. Photo-oxidation reactions should then occur mainly at the (010) interface, consistent with a recent suggestion,^29^ while the abundance of O_br_H groups on the (210) facet should favor the evolution of H_2_ on this facet. Although our simulations have clear size and time scale limitations and cannot describe nonadiabatic processes such as electron–hole recombination, the segregation mechanism identified in this work provides significant atomistic insight into the experimentally observed enhanced photocalytic properties of the nanorods.

## Conclusions

Ab initio molecular dynamics simulations of stoichiometric and reduced brookite nanorods in liquid water show that the presence of surface oxygen vacancies with their associated reduced Ti^3+^ ions is essential to achieve high reactivity for water dissociation over the entire surface of the nanorod. Importantly, the O_br_H and the terminal Ti–OH hydroxyl groups tend to accumulate on different facets of the nanorod, notably (210) and (010), respectively (Figure 3). While the large amount of Ti_4*c*_–OH^–^ groups promotes the localization of photoexcited holes on the (010) facets, the O_br_H groups on the (210) facets stabilize the reduced states that remain in the subsurface and the core of the nanostructure. The separation of bridging and terminal hydroxyls can also facilitate photoinduced charge carrier separation, which is a key factor in the improved photocatalytic properties of the nanorod, and it can further explain its sustained activity over time. These findings could open new paths in the design and tailoring of enhanced catalytic properties of nanomaterials.

## Methods

AIMD simulations were performed using density functional theory (DFT) in the generalized gradient approximation (GGA), as implemented in the CP2K software.^33^ All the molecular dynamics simulations were run in a singlet state with the exception of the results shown in Figure S1, where the reduced nanorod was considered in its 2S + 1 = 13 electronic state. All electronic properties, such as band gaps and Density of States (DOS), were evaluated by self-consistent single-point calculations of selected molecular dynamics snapshots using the hybrid B3LYP functional, which includes a 20% fraction of Hartree–Fock exchange.^34^ To describe excited electron–hole pairs, we performed single-point spin-constrained hybrid B3LYP calculations of the same selected molecular dynamics snapshots. For the stoichiometric system, with a singlet ground state, we constrained the excited-state solution to the triplet state to mimic the excited state with an electron in the conduction band and a hole in the valence band.^41,43–45^ For the reduced system, based on our choice of the high-spin ground state, we constrained the excited state to the lowest energy state of multiplicity 2*S* + 3, which ensures that the excited hole is in the valence band. All excited-state calculations were performed on the geometry of the corresponding ground state. For more details, see the relative section in the SI.

## Supplementary Material



## Notes

Time evolution of water dissociation of reduced brookite nanorod in a high spin state (of multiplicity 13); Water oxygen density profiles; Time evolution of the potential energies of the hydrated stoichiometric and reduced nanorods; Electron spin density of the reduced dry and wet nanorod; Excited electron–hole pair spin densities for the dry stoichiometric and reduced nanorods; Densities of states for the dry and hydrated stoichiometric and reduced nanorods (PDF)
