# Supplementary material for: Insights into the Reactivity of Brookite TiO2 Nanorods in Liquid Water from Ab Initio Molecular Dynamics Simulations
Source: ACS Catal. 2026 Feb 9;16(8):7177–84. doi: 10.1021/acscatal.5c09228 (PMC13097143; doi:10.1021/acscatal.5c09228)
Supplement: Supplementary file 1 [file cs5c09228_si_001.pdf]

Supporting Information: Insights into the Reactivity of Brookite TiO_2 Nanorods in Liquid Water from Ab Initio Molecular Dynamics Simulations

Lorenzo Agosta,[*,†,‡] Giuseppe Zollo,[¶] and Annabella Selloni[‡]

†*Department of Chemistry, Ångström Laboratory, Uppsala University, 751 21 Uppsala, Sweden*

‡*Department of Chemistry, Princeton University, Princeton, New Jersey, USA*

¶*Dipartimento di Scienze di Base e Applicate per l'Ingegneria, University of Rome "La Sapienza", Via A. Scarpa 14–16, 00161 Rome, Italy*

E-mail: lorenzo.agosta@kemi.uu.se

Methods and models

AIMD simulations were performed using Density Functional Theory (DFT) in the generalized gradient approximation (GGA) as implemented in the CP2K software.[1] Specifically, we used the BLYP functional[2,3] with D3 dispersion corrections,[4] together with GTH pseudopotentials‘,[5,6] and polarized double-ζ Gaussian basis set (DZVP)[7] for the valence electrons. This combination of DFT-GGA functional plus dispersion correction has been shown to describe the structure and dynamics of water at room temperature in good agreement with experiments.[8–10] The simulations were run with a time step of 0.5 fs in an NVT ensemble where the temperature was controlled by velocity rescaling[11] at 310 K. All the molecular dynamics simulations were run in a singlet state with the exception of the results shown in Figure S1, where the reduced nanorod was considered in its 2S +1 =13 electronic state.

All electronic properties, such as band gaps and Density of States (DOS), were evaluated by self-consistent single-point calculations of selected molecular dynamics snapshots using the hybrid B3LYP functional, which includes a 20% fraction of Hartree-Fock exchange.[12] For the reduced system, we focused on the high-spin ground state of multiplicity 2S + 1, where 2S is the number of excess electrons in the nanorod. The DOS curves of different systems were aligned at the deepest oxygen-atom states.

To describe excited electron-hole pairs, we performed single-point spin-constrained hybrid B3LYP calculations of the same selected molecular dynamics snapshots. For the stoichiometric system, with a singlet ground state, we constrained the excited-state solution to the triplet state to mimic the excited state with an electron in the conduction band and a hole in the valence band.[13–16] For the reduced system, based on our choice of the high-spin ground state, we constrained the excited state to the lowest energy state of multiplicity 2S + 3, which ensures that the excited hole is in the valence band. All excited-state calculations were performed on the geometry of the corresponding ground state.

The model of the brookite nanorod used in our study is periodic along its principal (z) axis and has a diameter of $\sim$ 1.2 nm (see Figure 1 of the main text). The nanorod exposes (210), (100), and (010) facets, with relative areas proportional to the corresponding surface energies[17,18] according to the Wulff construction. All facets expose 2-coordinated bridging oxygen atoms, O_{br}, as well as undercoordinated Ti atoms, notably five-fold coordinated Ti_{5c} atoms on the (100) and (210) facets and four-fold coordinated Ti_{4c} atoms on the (010) facet, supposedly the most reactive sites. To model hydrated nanorods, the simulation box was filled with liquid water at the experimental room temperature density of $\sim$ 1 g cm^{-3} (360 molecules).

To represent the reduced brookite nanorod, we created oxygen vacancies by removing bridging oxygen atoms on the surface of the nanorod. To identify the most probable sites for oxygen vacancies, we calculated the formation energy of an O_{br} vacancy at the O_1, O_2, and O_3 sites shown in Figure 1 of the main text. The formation energy of an oxygen vacancy was calculated from the expression:

$$\Delta E_{vac} = E_{nanorod} - E^{vac}_{nanorod} - E_{O_2}/2 \tag{S1}$$

where $E_{nanorod}$ and $E^{vac}_{nanorod}$ are the total energies of the stoichiometric and reduced nanorods,

respectively, and E_{O_2} is the total energy of an isolated oxygen molecule in its ground state (triplet). From Table S1, it appears that the most favorable site for an oxygen vacancy is O_1. We then created 6 (six) oxygen vacancies by removing two rows of O_1 bridging oxygen atoms along the nanorod axis. The locations of excess electrons generated by oxygen vacancies were identified by calculating the spin densities of selected molecular dynamics snapshots. The calculations were performed at the B3LYP level assuming that the (twelve) excess electrons in the reduced nanorod were in the high-spin (ferroelectric) configuration with multiplicity 13.

Table S1: Formation energies of surface O_{br} vacancies at the sites shown in Fig. 1 of the main text, referred to that of a vacancy at the O_1 site.

$\mathbf{O_2}$ **Vacancy**	**- Relative Energy (eV)**
O_1	0.0
O_2	2.3
O_3	2.2

In order to compute the water adsorption and the relative hydrated nanorods stability (see Figure S3) we used the following expression:

$$E_{ads} = (E^{red}_{nanorod+water} - E^{red}_{nanorod}) - (E^{sto}_{nanorod+water} - E^{sto}_{nanorod}) \quad \text{(S2)}$$

where $E^{red}_{nanorod+water}$ and $E^{sto}_{nanorod+water}$ are the mean potential energy values at equilibrium of the reduced and stoichiometric nanorods immersed in water, respectively. $E^{red}_{nanorod}$ and $E^{sto}_{nanorod}$ are the mean potential energy values at equilibrium at 300K of the reduced and stoichiometric nanorods immersed in vacuum, respectively. To note that the number of water molecules in the hydrated systems is the same.

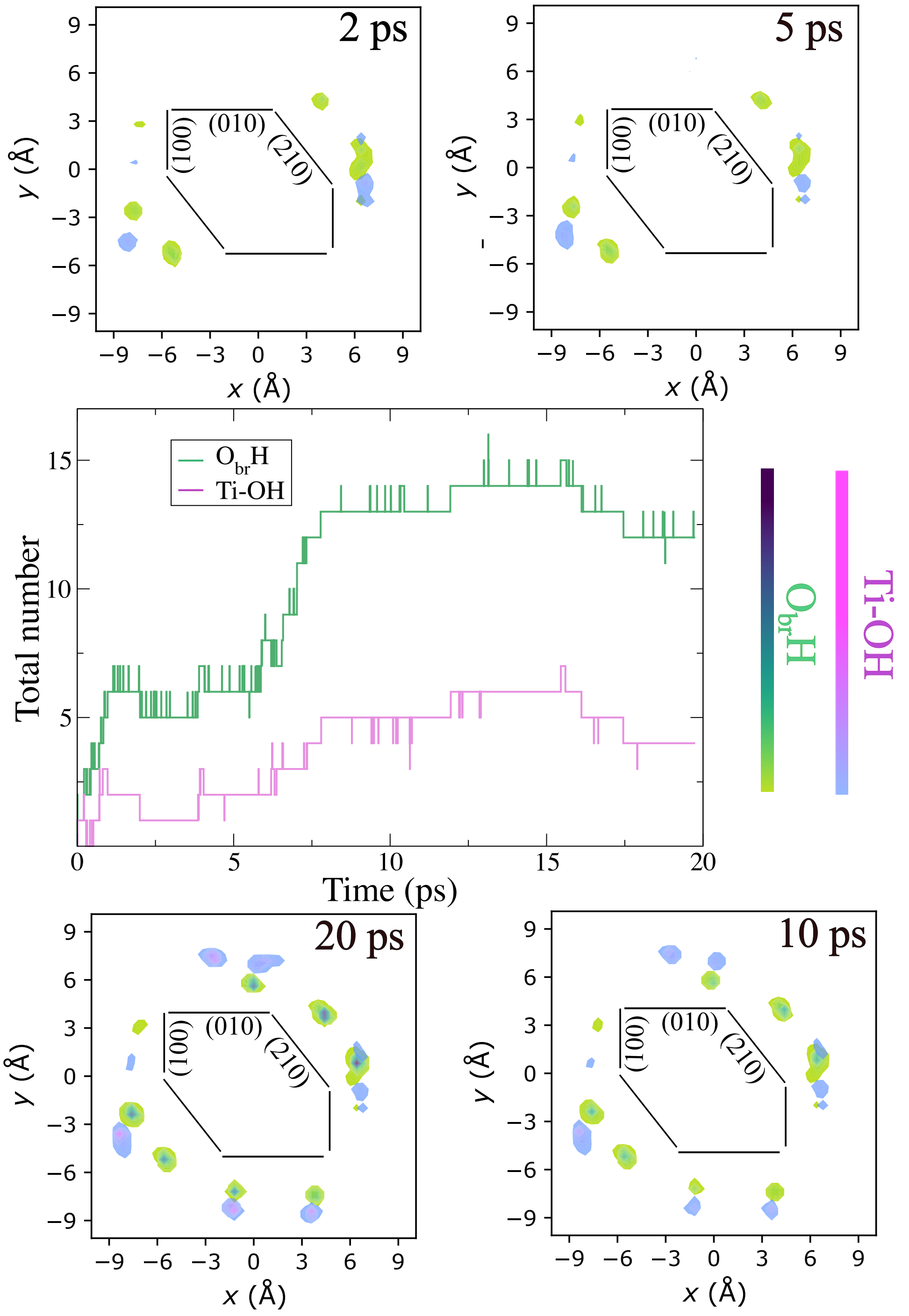

Figure S1: Time evolution of the amount of water dissociation on the different facets of the hydrated reduced brookite nanorod in a high spin state (of multiplicity 13) along a $\sim$ 20 ps AIMD simulations. The central panel shows the time evolution of the total number of Ti$-$OH and $O_{br}H$ groups on the surface of the nanorod. The surrounding panels show the distribution of Ti$-$OH and $O_{br}H$ groups on the various facets at selected times along the AIMD trajectory. The black lines indicate the positions of the facets.

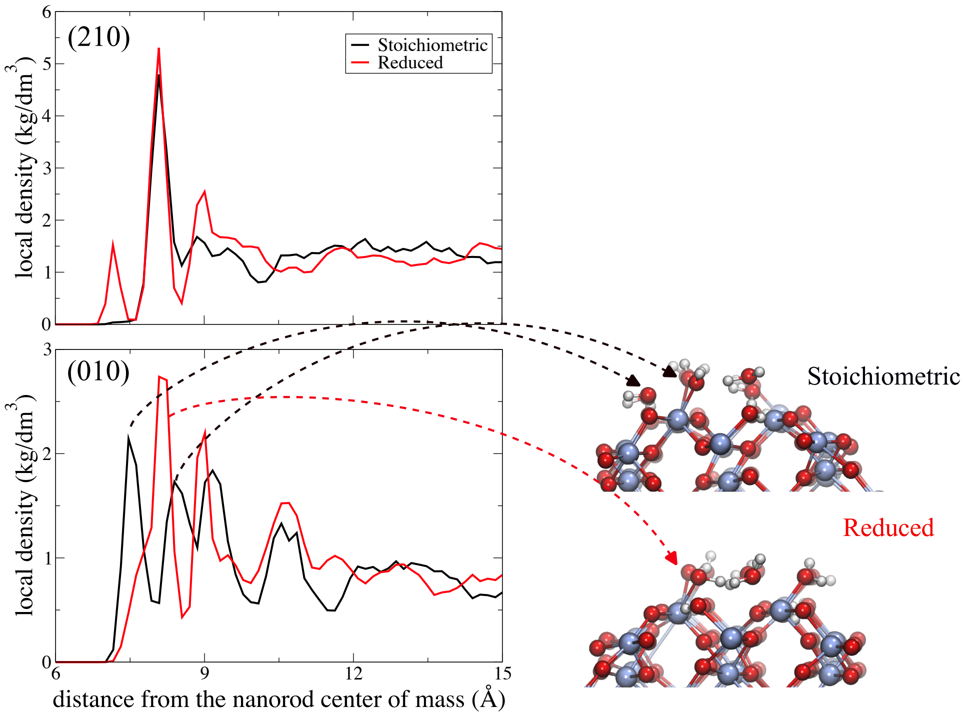

Figure S2: Water oxygen density profiles along the directions perpendicular to the (210) and (010) facets (upper and lower panel, respectively) of the stochiometric and reduced nanorods. Each profile is obtained by averaging the contributions of the two exposed (210) and (010) facets. On the (210) facet, the density peak at ~ 7 Å for the reduced nanorod is due to the $O_{br}H$ groups formed by the dissociative adsorption of water molecules at oxygen vacancies. Although there are no significant differences between the stoichiometric and reduced nanorods for adsorbed water at Ti_{5c} sites, the density of adsorbed water at $O_{br}H$ sites (peak at ~ 9 Å) is higher in the reduced case. On the (010) facet, the water molecules show two modes of adsorption at the Ti_{4c} sites (peaks at 7.5 and 8.7 Å) in the stoichiometric case, when only a few Ti−OH groups are present. In the reduced nanorod, these two peaks collapse in a single adsorption mode, as shown by the peak at 8.2 Å.

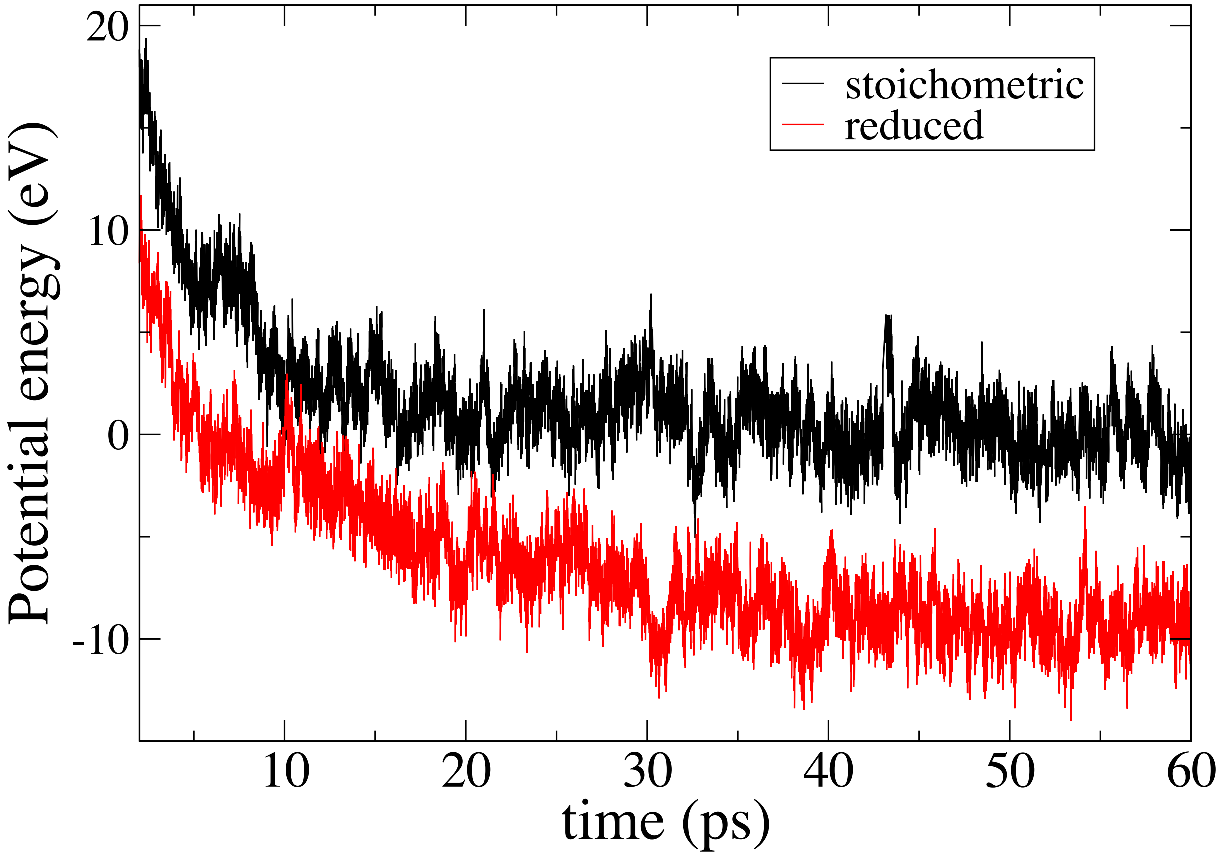

Figure S3: Time evolution of the potential energies of the hydrated stoichiometric and reduced nanorods. For each system the potential energy of the (nanorod + water) system is referred to the average potential energy of the corresponding (stoichiometric or reduced) dry nanorod at 300K. Given that the number of water molecules in the two systems is the same, this plot provides the difference in their water adsorption energies. The reduced nanorod is stabilized in bulk water at a much greater extent (about 9 eV) than its stoichiometric counterpart.

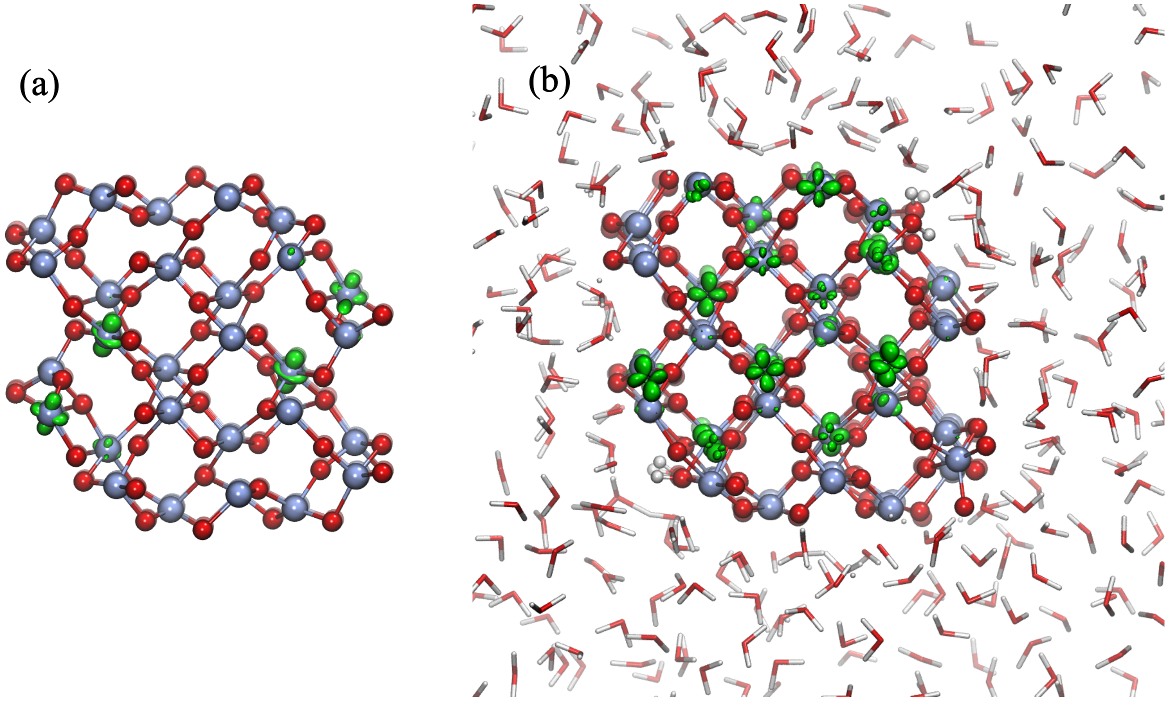

Figure S4: Electron spin density of the reduced nanorod (a) in its optimized T = 0 K geometry under vacuum condition, (b) immersed in water at 310 K. The configuration at room temperature is that of a representative snapshot. The light green iso-surfaces represent the unpaired electrons in the reduced state.

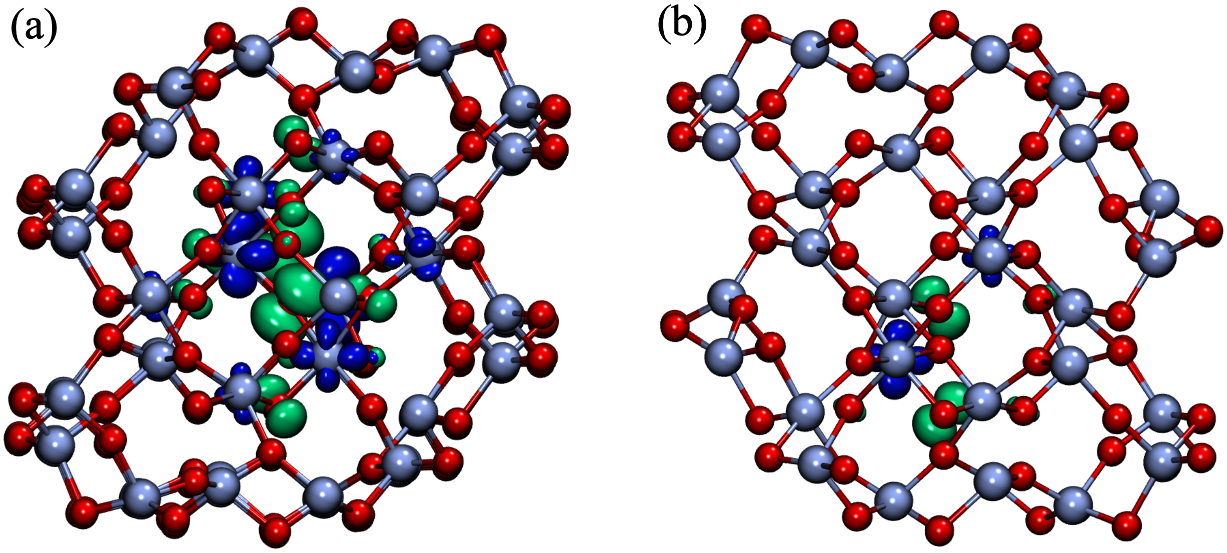

Figure S5: Excited electron-hole pair spin densities for the dry (a) stoichiometric and (b) reduced nanorods in the optimized ($T = 0$ K) geometries of the ground state. For the reduced nanorod, the reported density was obtained by subtracting the spin density of the ground state from that of the excited state calculated as described in Methods and Models. In both stoichiometric and reduced nanorods, the excited electron and hole are represented by the blue and green iso-surfaces respectively. The excited electron is localized in the Ti-3d states and the hole in the O-2p states of a few atoms in the core of the nanorod.

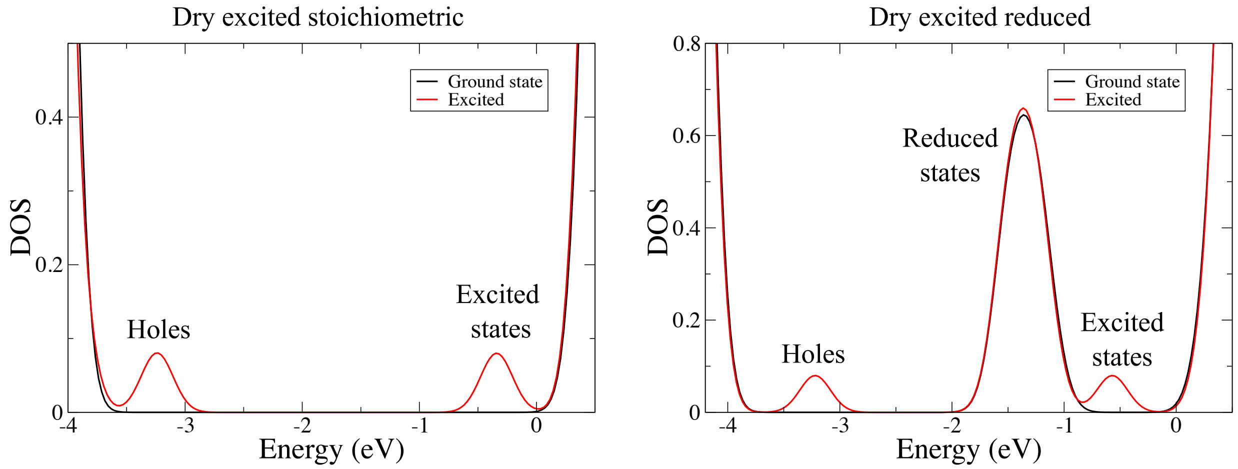

Figure S6: Densities of states for the dry stoichiometric (left panel) and dry reduced (right) nanorods in their ground (black) and excited (red) states, calculated using the hybrid B3LYP functional at the optimized (T = 0 K) geometry of the ground state. The peaks associated with the excited electrons (”excited states”) and holes (”holes”) are indicated, as well as the states of the excess electrons (”reduced states”) in the case of the reduced nanorod.

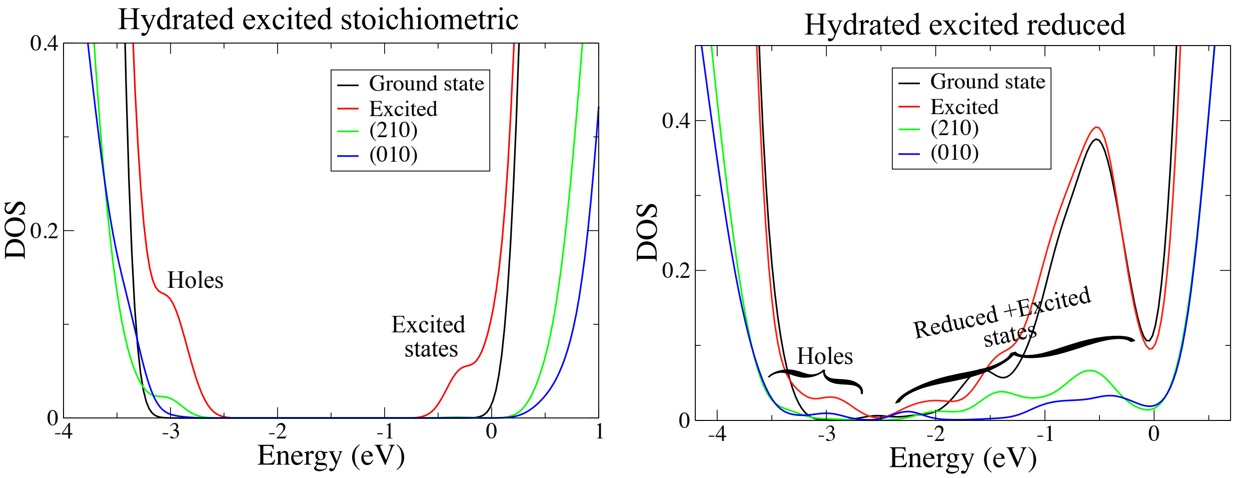

Figure S7: Densities of states for the hydrated stoichiometric (left) and reduced (right) nanorods in their ground (black) and excited (red line) states calculated using the B3LYP functional. Also shown are the projected DOS of the under-coordinated oxygen and titanium atoms belonging to the (210) and (010) facets (green and blue lines). The reported curves are the average of 7 and 15 snapshots for the stoichiometric and reduced case, respectively.

References

(1) Hutter, J.; Iannuzzi, M.; Schiffmann, F.; VandeVondele, J. CP2K: atomistic simulations of condensed matter systems. *Wiley Interdiscip. Rev.: Comput. Mol. Sci.* **2014**, *4*, 15.

(2) Becke, A. D. Density-Functional Exchange-Energy Approximation With Correct Asymptotic Behavior. *Phys. Rev. A* **1988**, *38*, 3098–3100.

(3) Lee, C.; Yang, W.; Parr, R. G. Development of the Colle-Salvetti Correlation-Energy Formula Into a Functional of the Electron Density. *Phys. Rev. B* **1988**, *37*, 785–789.

(4) Grimme, S. Semiempirical GGA-Type Density Functional Constructed with a Long-Range Dispersion Correction. *J.Comp.Chem.* **2010**, *132*, 154104–154119.

(5) Goedecker, S.; Teter, M.; Hutter, J. Separable Dual-Space Gaussian Pseudopotentials. *Phys. Rev. B* **1996**, *54*, 1703–1710.

(6) Krack, M. Pseudopotentials for H To Kr Optimized for Gradient-Corrected Exchange-Correlation Functionals. *Theoretical Chemistry Accounts* **2005**, *114*, 145–152.

(7) VandeVondele, J.; Hutter, J. Gaussian Basis Sets for Accurate Calculations on Molecular Systems in Gas and Condensed Phases. *J. Chem. Phys.* **2007**, *127*, 114105.

(8) Bankura, A.; Karmakar, A.; Carnevale, V.; Chandra, A.; Klein, M. L. Structure, Dynamics, and Spectral Diffusion of Water from First-Principles Molecular Dynamics. *The Journal of Physical Chemistry C* **2014**, *118*, 29401–29411.

(9) Agosta, L.; Brandt, E. G.; Lyubartsev, A. P. Diffusion and reaction pathways of water near fully hydrated TiO_2 surfaces from ab initio molecular dynamics. *J. Chem. Phys.* **2017**, *147*, 024704.

(10) Morawietz, T.; Singraber, A.; Dellago, C.; Behler, J. How van der Waals interactions determine the unique properties of water. *Proceedings of the National Academy of Sciences* **2016**, *113*, 8368–8373.

(11) Bussi, G.; Donadio, D.; Parrinello, M. Canonical Sampling Through Velocity Rescaling. *J. Chem. Phys.* **2007**, *126*, 014101.

(12) Becke, A. D. Density-functional thermochemistry. III. The role of exact exchange. *The Journal of Chemical Physics* **1993**, *98*, 5648–5652.

(13) Di Valentin, C.; Selloni, A. Bulk and Surface Polarons in Photoexcited Anatase TiO2. *The Journal of Physical Chemistry Letters* **2011**, *2*, 2223–2228.

(14) Migani, A.; Blancafort, L. Excitonic Interfacial Proton-Coupled Electron Transfer Mechanism in the Photocatalytic Oxidation of Methanol to Formaldehyde on TiO2(110). *Journal of the American Chemical Society* **2016**, *138*, 16165–16173, PMID: 27960348.

(15) Migani, A.; Blancafort, L. What Controls Photocatalytic Water Oxidation on Rutile TiO2(110) under Ultra-High-Vacuum Conditions? *Journal of the American Chemical Society* **2017**, *139*, 11845–11856.

(16) Daldossi, C.; Di Valentin, C.; Selloni, A. Pathways of Photocatalytic Oxidation of Formic Acid on Dry and Hydrated Anatase TiO2 Surfaces. *ACS Catalysis* **2025**, *15*, 11487–11501.

(17) Gong, X.-Q.; Selloni, A. First-principles study of the structures and energetics of stoichiometric brookite TiO_2 surfaces. *Phys. Rev. B* **2007**, *76*, 235307.

(18) Esch, T.; Gadaczek, I.; Bredow, T. Surface structures and thermodynamics of low-index of rutile, brookite and anatase - a comparative DFT study. *Appl. Surf. Sci.* **2014**, *288*, 275–287.
